# Collagen Film Activation with Nanoscale IKVAV-Capped Dendrimers for Selective Neural Cell Response

**DOI:** 10.3390/nano11051157

**Published:** 2021-04-28

**Authors:** Jessica J. Kim, Daniel V. Bax, Robert Murphy, Serena M. Best, Ruth E. Cameron

**Affiliations:** Department of Materials Science and Metallurgy, University of Cambridge, 27 Charles Babbage Road, Cambridge CB3 0FS, UK; jessicajkim1@gmail.com (J.J.K.); dvb24@cam.ac.uk (D.V.B.); rm645@cam.ac.uk (R.M.)

**Keywords:** bioactivated materials, collagen, dendrimers, IKVAV pentapeptide

## Abstract

Biocompatible neural guidance conduits are alternatives to less abundant autologous tissue grafts for small nerve gap injuries. To address larger peripheral nerve injuries, it is necessary to design cell selective biomaterials that attract neuronal and/or glial cells to an injury site while preventing the intrusion of fibroblasts that cause inhibitory scarring. Here, we investigate a potential method for obtaining this selective cellular response by analysing the responses of rat Schwann cells and human dermal fibroblasts to isoleucine-lysine-valine-alanine-valine (IKVAV)-capped dendrimer-activated collagen films. A high quantity of nanoscale IKVAV-capped dendrimers incorporated onto pre-crosslinked collagen films promoted rat Schwann cell attachment and proliferation, and inhibited human dermal fibroblast proliferation. In addition, while pre-crosslinked dendrimer-activated films inhibited fibroblast proliferation, non-crosslinked dendrimer-activated films and films that were crosslinked after dendrimer-activation (post-crosslinked films) did not. The different cellular responses to pre-crosslinked and post-crosslinked films highlight the importance of having fully exposed, non-covalently bound biochemical motifs (pre-crosslinked films) directing certain cellular responses. These results also suggest that high concentrations of nanoscale IKVAV motifs can inhibit fibroblast attachment to biological substrates, such as collagen, which inherently attract fibroblasts. Therefore, this work points toward the potential of IKVAV-capped dendrimer-activated collagen biomaterials in limiting neuropathy caused by fibrotic scarring at peripheral nerve injury sites.

## 1. Introduction

Nerve autografts, which are nerve tissues transplanted from one part of the body to another in the same individual, are the gold standard for severed peripheral nerve reconstruction [1,2]. However, autografts only effectively bridge small gaps (<5 cm) and are in limited supply [1]. Artificial nerve grafts, such as neural guidance conduits, that promote neural regeneration via biocompatible, activated scaffolds have been increasingly investigated as viable alternatives to more scarce autologous tissues [1,2]. One important requirement for viable neural guidance conduits is their ability to impede fibroblast infiltration. Extensive fibrotic scar tissue can form at an injury-site before neurites and glial cells can rebuild neural tissue [3,4,5]. This fibrosis can prevent full-functional recovery for larger injuries and may result in debilitating neuromas (“pinched nerves”) [5]. Fibrosis is an especially pervasive problem because initial fibrosis naturally stiffens the extracellular matrix in tissue surrounding an injury-site. The decreased elasticity creates an environment that can transform the phenotype of nearby cells into fibroblasts, providing a positive feedback loop that perpetuates fibrosis [4,6]. While fibroblasts stabilise peripheral nerve wounds and play important roles in glial Schwann cell migration for proper axonal regrowth [5,7], excessive fibrotic scarring is inhibitory for downstream neural repair [4]. Most commercially available neural guidance conduits are hollow tubes that prevent fibroblast infiltration due to their structure [8]. Given that larger nerve gaps require neurites and glial cells to spread across a larger surface area for a longer period of time, it is especially important to design biomaterials that reduce fibroblast adhesion and proliferation via inherent biochemical interactions in addition to structural defence.

Attaching small biochemical motifs to a larger scaffold is an attractive method to engage a specific subset of cells on biocompatible materials for neural applications [9]. Small peptides such as YIGSR (tyrosine-isoleucine-glycine-serine-arginine) and IKVAV are laminin-derived sequences that promote the proliferation of a variety of neural cells [9,10], including glial Schwann cells [11]. Specifically, Hosseinkhani and co-workers have shown that IKVAV peptides attached to collagen surfaces improve the cellular responses of dorsal root ganglions [12]. However, IKVAV has only been shown to minimise fibroblast binding to polyimide, SiO_2_ and gold surfaces [13]. It is unknown whether IKVAV peptides bound to collagen can promote the proliferation of glial cells, which support neurite regrowth [4], while simultaneously inhibiting the growth of fibroblasts, which can inhibit proper neuronal repair [4]. Understanding these effects, to attain the desired cellular selectivity, could better inform design principles for fabricating biomaterials more suited for next generation peripheral nerve repair.

An important consideration when activating collagen scaffolds with small molecules is to ensure that appended biochemical motifs are physically accessible to cells. Recent research has looked into the use of poly ε-lysine dendrimers, which are branched peptides, to activate different scaffold materials such as collagen [10,14,15,16,17,18,19]. Terminal amines on dendrimers can be capped with various biochemical motifs, such as IKVAV, to present bioactive groups to cells in a spatially optimised, three-dimensional manner. Previous studies by Duan and co-workers showed that YIGSR-capped dendrimers incorporated into three-dimensional collagen gels show favourable cellular responses of corneal epithelial cells [10,18,19]. Studies by Perugini and co-workers have shown that YIGSR-capped dendrimers also allow human mesenchymal stem cells to organise into spheroids, which is their configuration in vivo [14,15]. While two-dimensional linear peptides have limited spatial orientations, appending linear peptides to the termini of larger branched frameworks can increase the functional quantity of biochemical motifs that are optimally oriented to elicit a cellular response.

Given the potential of dendrimer-activated biomaterials for improved neural repair, the objective of this work is to integrate nanoscale IKVAV-capped poly ε-lysine dendrimers onto collagen surfaces and study the response of rat Schwann cells (RSCs) and human dermal fibroblasts (HDFs). By adding IKVAV-capped dendrimers to non-crosslinked collagen films, pre-crosslinked collagen films or by crosslinking collagen films after dendrimer addition (post-crosslinking), the response of Schwann cells and fibroblasts to different surface presentations of dendrimers can be studied. Previous studies have shown that non-crosslinked collagen films possess a lower tensile modulus compared to 1-ethyl-3-(3-dimethylaminopropyl)carbodiimide hydrochloride (EDC)/ N-hydroxy-succinimide (NHS) crosslinked collagen films, thus providing different environments for cellular attachment and proliferation [20]. What is less known is how EDC/NHS crosslinking can affect the presentation of IKVAV-capped dendrimers and affect downstream cellular interactions. Thus, both “pre-crosslinked” and “post-crosslinked” collagen films were fabricated to probe such interactions. In general, understanding how these materials specifically attract Schwann cells, and inhibit fibroblast proliferation can provide important insight into designing biomaterials that limit neuropathy brought on by excessive fibrotic scarring after synthetic material implantation.

## 2. Materials and Methods

### 2.1. Materials

Insoluble bovine dermis type I collagen powder (product code: 01AWB003) was obtained from Devro PLC, (now Collagen Solutions, Glasgow, UK). 16-arm IKVAV-capped poly ε-lysine dendrimers (lot numbers: H41511K17-04, H41511K17-17) were obtained from Tissue Click Ltd., Brighton, UK. Human dermal fibroblasts (HDFs) were sourced from Sigma Aldrich (product code: C-12300, Gillingham, UK). RSC-96 rat Schwann cells (RSCs) were obtained from the ATCC cell biology collection (product code: ATCC^®^ CRL-2765™, Manassas, VA, USA). Unless stated otherwise, all other reagents were analytical grade as obtained from Sigma-Aldrich, Gillingham, UK.

### 2.2. Collagen Film Preparation

Collagen slurries were prepared by swelling a 0.5% (*w*/*v*) suspension of collagen powder in 50 mM acetic acid at 4 °C for 3 days. Using a commercially available blender, the hydrated powder was blended for 2 min, rested for 2 min, and blended again for another 2 min at 22,000 rpm. Air bubbles were removed from the suspension by centrifuging at 2500 rpm for 30 s. After cooling the suspension at 4 °C overnight, films of micron-scale thickness were cast by reverse pipetting 200 µL of slurry per well into a CytoOne TC-treated 48-well plate (STARLAB, Milton Keynes, UK) and drying for 48 h in a laminar flow cabinet [21,22].

### 2.3. Dendrimer Fluorescence Tag Conjugation

To visualise the dendrimers on collagen films, α-amines on terminal valines and/or ε-amines on lysines were tagged with Atto 565 nm NHS ester fluorescent probes (molecular weight (MW) = 708.11 Da). Atto 565 nm NHS ester stock solution was prepared by dissolving 1 mg of powdered ester dye in 0.5 mL of dry dimethyl sulfoxide (DMSO). One mg of dendrimer powder was suspended in 1 mL of phosphate-buffered saline (PBS). For dye conjugation, it was assumed that a 1:1 molar ratio of dye to free primary amine would tag each terminal amine with Atto dye. To adjust to pH 9, 1M NaHCO_3_ was added according to the manufacturer’s instructions.

To remove non-conjugated dye, the agitated solutions were dialysed for 24 h in Slide-A-Lyzer dialysis cassettes (Thermo Fischer, Paisley, UK). The concentrations of purified dye-conjugated dendrimers were measured colourimetrically with a Pierce Bicinchoninic Acid (BCA) Protein Assay Kit, (Cat. No: 23227; Thermo Fischer, Paisley, UK) using a Bovine serum albumin (BSA) protein standard. Prior to measurement, the mixtures were agitated on a rocker for 30 s covered in foil and then incubated at 37 °C for 40 min (78% dye-conjugated dendrimers) or agitated on a rocker for 20–30 min at room temperature (8% dye-conjugated dendrimers). Absorbance values were read at 562 nm (A_562_) immediately after incubation using a Fluostar Optima plate reader (BMG Labtech, Ortenberg, Germany). The absorbance values of the control samples were subtracted from the absorbance values of the experimental samples to correct for background dye absorption.

### 2.4. Incorporation of Dendrimers into 2D Collagen Films

Dye-conjugated IKVAV-capped dendrimers were added to non-crosslinked films (A), pre-EDC/NHS crosslinked films (B), or non-crosslinked films that were crosslinked with EDC/NHS agents after dendrimer addition (C). The three varieties of dendrimer-activated collagen films were simultaneously fabricated over three days to ensure that they were exposed to the same experimental conditions. To coat the collagen films, which were used as dried, with dye-conjugated dendrimers, 120 µL of dendrimer solution (µg of dendrimer/mL of PBS) was added to non-crosslinked (A,C) or crosslinked (B) collagen films. The films were incubated for 1.5 h on a rocker at room temperature covered in foil. After incubation, excess dendrimer solution was removed, and the films were washed with 3 × 200 µL PBS. The films were dried in a dark fume hood overnight.

To chemically and covalently crosslink the collagen films, a 5:2:1 molar ratio of EDC/NHS/COO-(Col) in 75% (*v*/*v*) ethanol was added and incubated for 1.5 h. These conditions were deemed optimal by Olde Damink and co-workers [23] for standard (100%) crosslinking. Non-crosslinked films (A) were incubated in 75% ethanol without any crosslinking agents for 1.5 h. After incubation, the crosslinking solution was removed, and the films were washed 3 × 200 µL deionised water. All films were dried in a dark fume hood overnight. Fully fabricated films were kept at 4 °C until use.

### 2.5. Dissociation of Dendrimers from 2D Collagen Films

To probe dendrimer-collagen interactions, all samples were washed with various detergents for increasing lengths of time. The samples were washed with PBS, 75% ethanol (EtOH) (*v*/*v*) in deionised water, 0.1% Tween (*v*/*v*) in PBS, saturated saline, or 0.1 M citrate buffer (pH 3). Collagen films were analysed at 0 h (pre-wash), and after 2 h, 1 day, 2 days, 3 weeks, and 7 weeks. Fluorescent images of the collagen films were taken with a Zeiss Axio Observer Z1 microscope fitted with an Axiocam 530 camera (Oberkochen, Germany) after each time interval. Fluorescent images were taken at 10× magnification and at 565 nm and 488 nm to detect the dye-conjugated dendrimers and collagen autofluorescence, respectively. Prior to imaging, the samples were washed with 3 × 200 µL deionised water, and all images were taken of collagen films that were hydrated in 200 µL of either fresh PBS or deionised water. In between imaging time points, the samples were incubated in their respective washing solutions on a rocker at room temperature covered in foil. ImageJ software was used to quantify the dendrimers bound to the collagen films after each time interval.

### 2.6. Cell Attachment Assay

RSCs and HDFs were cultured in Dulbecco’s modified Eagle’s medium (DMEM) containing 10% (*v*/*v*) foetal bovine serum and 1% (*v*/*v*) streptomycin/penicillin antibiotics in a humidified incubator with 5% CO_2_ at 37 °C. RSCs were passaged 1:20 and HDFs were passaged 1:4 every 3–4 days. Cell layers at ~75% confluence were harvested for analysis by detaching the cells from T75 flasks with 0.05% (*w*/*v*) trypsin/0.02% (*w*/*v*) EDTA, centrifuging the cells at 180 g for 3 min, and resuspending the cells in serum-free DMEM.

Before seeding the cells, the dendrimer-activated collagen films were blocked for 1.5 h with 300 µL of 2% (*v*/*v*) BSA solution in PBS and washed with 3 × 300 µL PBS to prevent non-specific cell binding. The cells were seeded at a density of 5 × 10^5^ cells/mL for RSCs and 3.06 × 10^5^ cells/mL for HDFs. These cell densities were determined using a haemocytometer. Three hundred µL of appropriately diluted cells were added to the films and tissue culture plastic (TCP) controls and incubated for 1.5 h in a humidified incubator with 5% CO_2_ at 37 °C. Loosely bound cells were gently removed with 3 × 300 µL PBS washes.

The number of cells bound to the collagen films were quantified colourimetrically by measuring the amount of lactate dehydrogenase (LDH) released from lysed cells into the media [20,24]. To lyse bound cells, 100 µL of 2% Triton (*v*/*v*) in deionised water was added to all the samples and incubated at 4 °C overnight. According to manufacturer recommendations, 100 µL of LDH substrate (Cat. No: 11644793001; Sigma Aldrich, Gillingham, UK) was made and was added to the samples and left to incubate until a significant colour change was apparent (20–30 min). Absorbance values were read at 490 nm (A_490_) using a Fluostar Optima plate reader (BMG Labtech, Ortenberg, Germany).

### 2.7. Cell Proliferation Assay

Dendrimer-activated collagen films were blocked with BSA solution and seeded with cells as for cell attachment analysis, except the cells were resuspended in media containing 10% (*v*/*v*) foetal bovine serum at a density of 1.46 × 10^4^ cells/mL and 2.2 × 10^4^ cells/mL for RSCs and HDFs, respectively. Eight hundred µL of these cell suspensions were incubated on the collagen samples for 72 h in a humidified incubator with 5% CO_2_ at 37 °C to allow for ~75% confluent proliferation.

### 2.8. Fixed Cell Imaging with Fluorescence Microscopy

To image the cell number on dendrimer-activated collagen films, the cells were fixed by adding 200 µL of 25% (*w*/*v*) glutaraldehyde directly to the cell media (final glutaraldehyde concentration of 5% (*w*/*v*)) for 30 min at room temperature. After the glutaraldehyde solution was removed, the films were washed 3× with sterile deionised water. The cells were stained with 200 µL of 5 µM DAPI (4′,6-diamidino-2-phenylindole) in water. The DAPI was incubated for 10–20 min at room temperature and then removed. The collagen films were washed 3× with sterile deionised water before they were imaged with a Zeiss Axio Observer Z1 microscope fitted with an Axiocam 530 camera. Fluorescent images were taken at 10× magnification and at 358 nm excitation wavelength to detect the DAPI stain. The number of cells per field of view were quantified with CellProfiler (version 3.1.9, Broad Institute, USA—open source).

### 2.9. Statistical Analysis

The data were analysed for normality and equal variances using Shapiro-Wilk (or Kolmogorov-Smirnov) and Levene’s tests, respectively, with *p* ≤ 0.05. When Levene’s test was statistically significant (*p* ≤ 0.05), a 2-way analysis of variance (ANOVA) was still conducted when sample sizes were roughly equivalent. Mean values were compared using 2-way ANOVA with Tukey’s post-hoc tests to determine statistical significance (*p* ≤ 0.05). Bonferroni comparisons were used for multiple comparisons. The values reported are means of triplicate measures ± standard deviations, unless stated otherwise. Error bars indicate standard deviations from the mean. N/S denotes *p* ≥ 0.05.

## 3. Results

### 3.1. Dye-Tagging Dendrimers for Visualisation on Collagen Substrates

**Figure 1:**
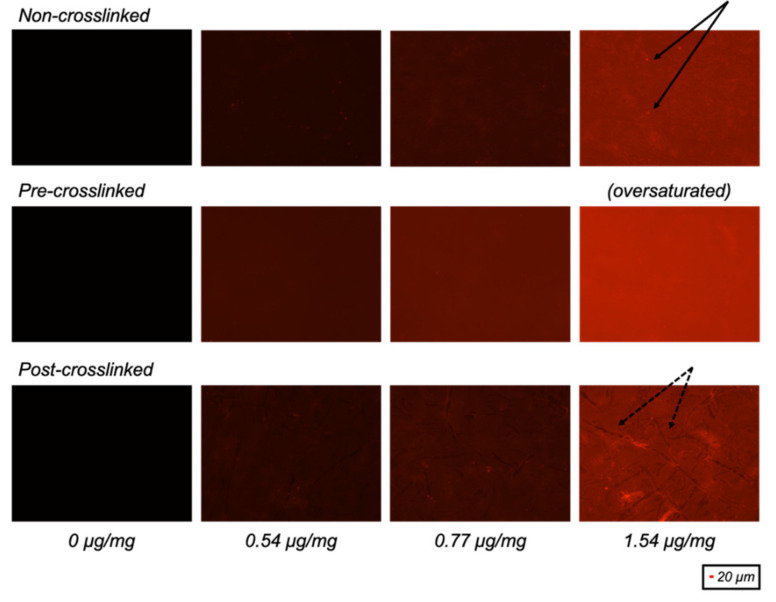
Fluorescence images of non-crosslinked, pre-crosslinked and post-crosslinked collagen films with different quantities of 78% dye-conjugated dendrimer activation (µg dendrimer per mg collagen). Presence of overexposed bright particulates (solid) and underexposed collagen “coils” (dashed) are indicated by black arrows.

To visualise and quantify the IKAV-capped dendrimers integrated onto collagen substrates, the dendrimers were first conjugated with Atto 565 nm fluorescent probes. The efficiency of Atto dye conjugation was investigated and the dendrimer:dye ratio was optimised. The percent conjugation was defined as the ratio of the molecular weight of the free 16-arm dendrimer (MW estimated to be ~10,250 Da based on the syntheses described by Perugini and co-workers) [14,15] to the molecular weight of the dye-conjugated 16-arm dendrimer. It was assumed that each dye molecule would conjugate one free terminal amine, wherein each dendrimer contains 32 free terminal amines. The dendrimer:dye ratio was kept above 1:1 to ensure that some terminal amines would remain untagged with dye and could thus serve as sites for EDC/NHS crosslinking to collagen (necessary for the post-crosslinked dendrimer-activated collagen films). The quantity of Atto dye-tagged dendrimers integrated onto each collagen film was optimised by analysing the concentration at which the dye-tagged dendrimers aggregated on the collagen substrates. An optimal concentration of dendrimer was selected so that the fluorescence intensity could be distinguished between the non-crosslinked, pre-crosslinked, and post-crosslinked films without the presence of particulates (overexposure) or collagen “coils” (underexposure), in the case of the post-crosslinked films (Figure 1). This was to ensure that a surface with even dye distribution and uniform intensity was analysed.

### 3.2. Association of Dye-Tagged Dendrimers with 2D Collagen Films

Dye-tagged IKVAV-capped dendrimers were incorporated onto collagen substrates using three different methods to yield three different types of collagen surface environments. Dye-tagged IKVAV-capped dendrimers were coated onto (1) non-crosslinked collagen films, (2) pre-crosslinked films that were initially crosslinked with EDC/NHS, and (3) non-crosslinked films that were subsequently crosslinked with EDC/NHS after the addition of dendrimers to yield “post-crosslinked” films. It was thought that crosslinked surfaces would be more hydrophobic given the ablation of exposed free amines (lysine-based) and carboxylates (glutamate and aspartate-based) upon EDC/NHS treatment [23,25]. Conversely, non-crosslinked surfaces were thought to be charged, given the presence of free terminal amines and carboxylates in native collagen [26,27,28]. Thus, these collagen film varieties were fabricated to understand how dendrimers, which could interact both non-specifically via steric bulk or, specifically, via charged amine termini, adsorbed onto the collagen surfaces. Given that cellular responses could be affected by different dendrimer-collagen chemical interactions, it was important to understand how the dendrimers associate and dissociate from the various collagen substrates to optimise these biomaterials for neural applications.

Dendrimers with low extent of Atto 565 nm dye-conjugation (8%) and high extent of dye-conjugation (78%) were investigated in this study. Of the dye-tagged dendrimers, 8% have a lower molecular weight and higher overall charge (fewer dye-consumed NH_3_^+^ groups) than 78% dye-tagged dendrimers, and it was hypothesised that differences between their interactions with the collagen films could be attributed to differences in steric bulk and overall charge.

**Figure 2:**
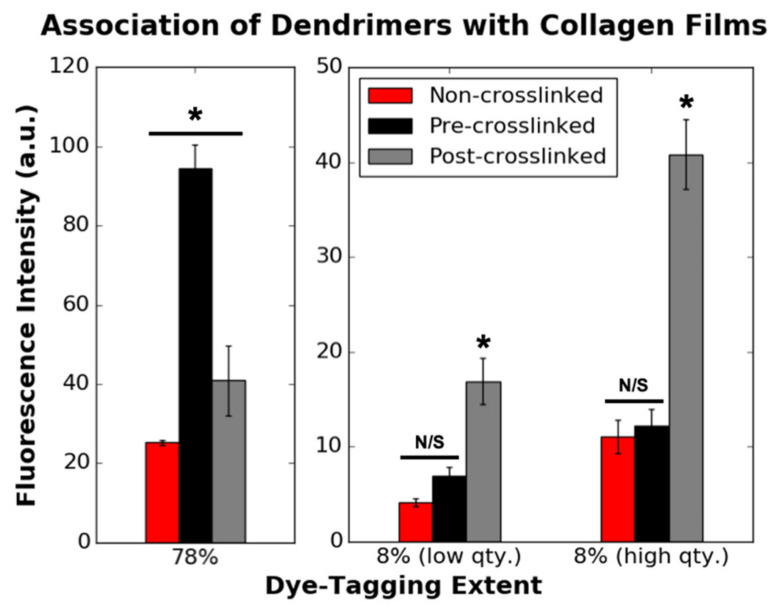
Initial association of 78% (0.77 µg/mg loading concentration) (*n* = 4) and 8% (*n* = 12) dye-tagged dendrimers with non-crosslinked, pre-crosslinked and post-crosslinked films. Eight percent (low qty.) and 8% (high qty.) correspond to 0.74 and 1.49 µg/mg loading concentrations (µg dendrimer per mg collagen), respectively. This data corresponds to the 0 h values in Figure 3 and Figure 4. N/S denotes *p* ≥ 0.05. * indicates statistically significant difference (*p* ≤ 0.001) from the two other substrate varieties with dendrimers with equivalent dye-tagging extents.

Figure 2 depicts the initial quantity of 78% and 8% dye-tagged dendrimers attached to various collagen substrates prior to incubation in various wash buffers. Similar loading concentrations of 78% and 8% dye-tagged dendrimers were used to ensure that the pattern of association was not dependent on loading concentration. Of the dye-tagged dendrimers, 78% had a higher initial association to pre-crosslinked films than non-crosslinked or post-crosslinked films (which were non-crosslinked at the point of dendrimer addition). Of dye-tagged dendrimers, 8% had higher initial association to post-crosslinked films and similar, yet lower, initial associations to non-crosslinked or pre-crosslinked films. From these results it was apparent that the charge densities on the two dye-tagged dendrimer variants, as well as the collagen surface properties, affected initial dendrimer binding to collagen.

### 3.3. Dissociation of Dye-Tagged Dendrimers from 2D Collagen Films

Long-term interactions between charged, dye-tagged IKVAV-capped dendrimers and non-crosslinked, pre-crosslinked, and post-crosslinked collagen films were probed by studying how dendrimers dissociate from the films when exposed to various solvents. As mentioned in the methods above, the solvents selected were PBS, 75% ethanol (EtOH) (*v*/*v*) in deionised water, 0.1% Tween (*v*/*v*) in PBS, saturated saline, and 0.1 M citrate buffer (pH 3). These solvents were chosen to probe different types of chemical interactions that could exist between the dendrimers and the surface of the collagen films. To probe non-ionic interactions, 75% EtOH was used, and saline and PBS solutions, which are salt-based buffers, were used to probe ionic interactions. To probe ionic and non-specific, non-covalent interactions, 0.1% Tween in PBS—a salt-based buffer containing a non-ionic detergent—was used. Citrate buffer (pH 3) was used to probe interactions at an acidic pH. It was thought that by incubating the films in wash buffer for an extended period of time and taking fluorescence measurements of the films at various time points, the relative rates of dendrimer dissociation from the collagen substrates could be compared. These trends would provide insight into how bulky, charged nanomaterials, such as dendrimers, interact with different collagen surface chemistries long-term, and how this could ultimately impact cell binding and proliferation.

Dissociation profiles of 8% and 78% dye-tagged IKVAV-capped dendrimers from the collagen films were obtained by washing the dendrimer-activated collagen films with the aforementioned solvents for increasing lengths of time. Collagen autofluorescence, measured at a different wavelength (488 nm) to dye-conjugated dendrimers (565 nm), was initially measured at each time point to ensure that collagen substrate breakdown was not a confounding factor for dendrimer dissociation (Supplementary Materials, Figures S1–S3). After confirming that non-crosslinked films were comparably stable to crosslinked films when incubated in aqueous wash buffers, dendrimer dissociation was analysed.

**Figure 3:**
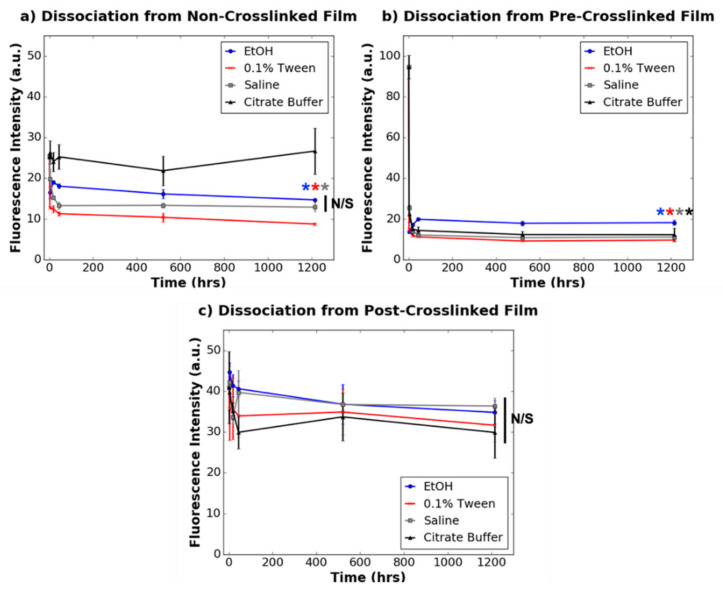
Dissociation of 78% dye-tagged dendrimers (0.77 µg/mg loading concentration) from (**a**) non-crosslinked films, (**b**) pre-crosslinked films, and (**c**) post-crosslinked films (*n* = 3 for all time points except for time point 0, which was *n* = 4). N/S denotes *p* ≥ 0.05. * denotes statistically significant difference (*p* ≤ 0.01) between the data point annotated (colour-coded) and the respective 0 h value for each collagen substrate. Statistical significance is only denoted for the final time point for clarity.

**Figure 4:**
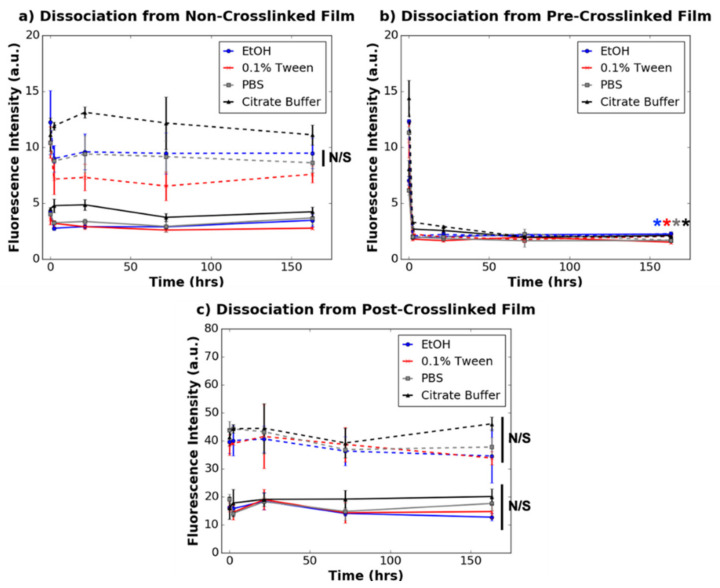
Dissociation of 8% dye-tagged dendrimers from (**a**) non-crosslinked films, (**b**) pre-crosslinked films, and (**c**) post-crosslinked films. Solid and dotted lines correspond to 0.74 µg/mg and 1.49 µg/mg dendrimer loading concentrations, respectively. N/S denotes *p* ≥ 0.05. * denotes statistically significant difference (*p* ≤ 0.01) between the data point annotated (colour-coded) and the respective 0 h value for each collagen substrate. Statistical significance is only depicted for 1.49 µg/mg dendrimer quantities for non-crosslinked and pre-crosslinked films and only for the final time point for clarity.

Figure 3 and Figure 4 depict the dissociation of 78% and 8% dye-tagged dendrimers, respectively, from non-crosslinked, pre-crosslinked and post-crosslinked films. For non-crosslinked films activated with 78% dye-tagged IKVAV-capped dendrimers, 0.1% Tween (in PBS) dissociated the greatest quantity of dendrimers, followed by saline and 75% ethanol, over a 1200 h time period. Citrate buffer did not significantly dissociate 78% dye-tagged dendrimers from the non-crosslinked collagen films (*p* ≥ 0.05) unlike the other wash buffers. For the non-crosslinked films activated with 8% dye-tagged dendrimers, none of the wash buffers significantly dissociated dendrimers from the films (*p* ≥ 0.05). These results suggest that the overall charge on the dendrimers affects how they interact with non-crosslinked collagen substrates over time.

Unlike the non-crosslinked films, for which dendrimer dissociation was dependent on both the extent of dye-conjugation and also the type of solvent used to dissociate the dendrimers, dendrimer dissociations from pre-crosslinked and post-crosslinked films were unaffected by these parameters. When dendrimer-activated pre-crosslinked films were washed with any of the wash buffers, there was drastic dissociation of both 8% and 78% dye-tagged dendrimers in <2 h (*p* ≤ 0.001), with limited dissociation for the remaining incubation time period. On the other hand, there was no significant dissociation (*p* ≥ 0.05) of either 8% or 78% dye-tagged IKVAV-capped dendrimers incorporated into post-crosslinked films when washed with any of the buffers. These results provide evidence that dendrimer-collagen interactions in the pre-crosslinked films are easily disturbed with a variety of aqueous solvents, and that dendrimer-collagen interactions in the post-crosslinked films are too strong to be disturbed by aqueous, ionic wash buffers.

As shown in Figure 4, it is evident that for all three substrate varieties, dendrimer-collagen dissociations were not dependent on loading concentration, as a two-fold higher concentration of 8% dye-conjugated dendrimers showed the same trends as described above. However, for the higher loading concentration of 8% dye-conjugated dendrimers used, some of the films displayed some particulates.

Overall, these interactions suggest that dendrimers likely interact with non-crosslinked films (which contain exposed carboxylate motifs) [28] via electrostatic or ionic interactions, given that different wash buffers had different effects on dendrimer dissociation. While 8% dye-tagged dendrimers did not dissociate to a significant extent using salt-based buffers, their higher density of charged amines, relative to 78% dye-tagged dendrimers, could provide greater points of contact with the collagen films, permitting association in ionic buffers. In addition, these results support that pre-crosslinked films (which do not contain exposed charged motifs, but rather inactive amides) [28,29] interact with collagen films via van der Waals non-covalent interactions as these dendrimers easily dissociated from the films in aqueous solution. Finally, it is likely that dendrimers interact with post-crosslinked films via amide linkages given that EDC/NHS agents were added to the films after dendrimer addition and subsequent aqueous salt-buffer washes could not remove the dendrimers from the collagen surfaces. These stronger chemical interactions between the charged IKVAV-capped dendrimers with the non-crosslinked and post-crosslinked films could explain why dendrimers remain bound to these substrate varieties for a longer period of time than dendrimers which form Van der Waals interactions with pre-crosslinked films. These results also suggest that despite the bulky steric profile of dendrimers, their branched conformation do not allow them to entangle onto the pre-cast collagen surface to a sufficiently permanent extent.

### 3.4. RSC and HDF Attachment to Dendrimer-Activated Collagen Films

After the mode of chemical interaction between the dendrimers and the various collagen films were probed, cellular responses to the various dendrimer-activated collagen films were studied. Rat Schwann cells (RSCs) were used to model how glial cells, which nourish neurites during regrowth [4], respond to IKVAV-activated collagen substrates. Human dermal fibroblasts (HDFs) were used to model how these inhibitory scar-forming cells interact with these substrates. For these studies, non-dye-tagged IKVAV-capped dendrimers were used.

**Figure 5:**
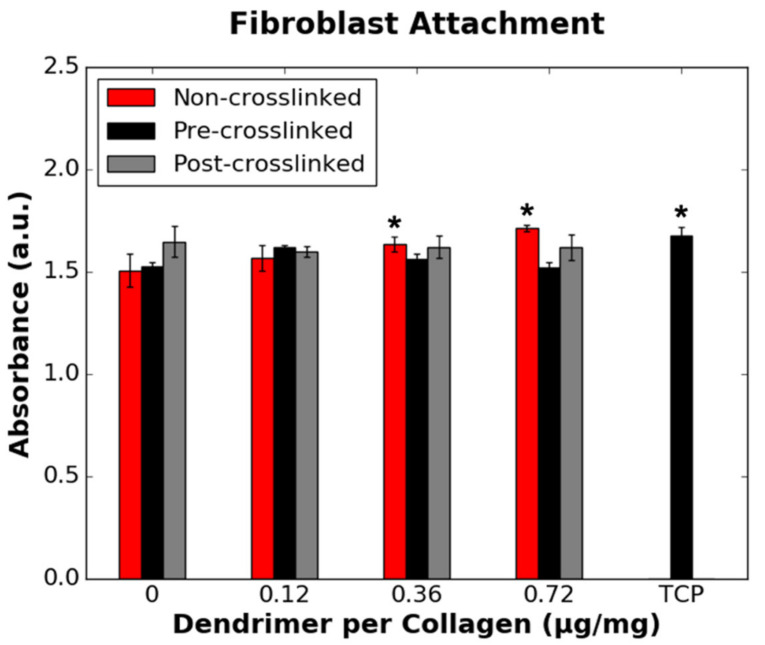
Effect of the quantity of IKVAV-capped dendrimers added to the various collagen films on HDF binding. HDFs were seeded onto non-collagen coated wells (TCP controls) on the same 48-well plate as pre-crosslinked films. * denotes statistically significant difference (*p* ≤ 0.05) between the data point annotated and the respective 0 µg/mg value for each collagen substrate.

To probe cellular adhesion, a modified LDH assay, as previously reported, was employed [20,24]. While a conventional LDH assay is often used to detect apoptotic cells via LDH release into the surrounding media, in this study, bound cells were deliberately lysed with 2% (*v*/*v*) Triton-X 100 in water. As all cells were lysed, the quantity of bound cells could be determined given a linear relationship between LDH detection and cell density [24]. Using this approach to quantity cellular adhesion, it was shown that the quantity of IKVAV-capped dendrimers (µg dendrimer per mg collagen) on the three collagen substrates had minimal effect on HDF binding, as shown in Figure 5. HDF adhesion was only increased for non-crosslinked films with 0.36 and 0.72 µg/mg dendrimer quantities, relative to non-crosslinked films without dendrimer activation. As HDFs attach to crosslinked and non-crosslinked films both with and without dendrimer activation, their binding may be dominated by their native propensity to bind collagen, regardless of the presence, or lack thereof, of IKVAV motifs.

**Figure 6:**
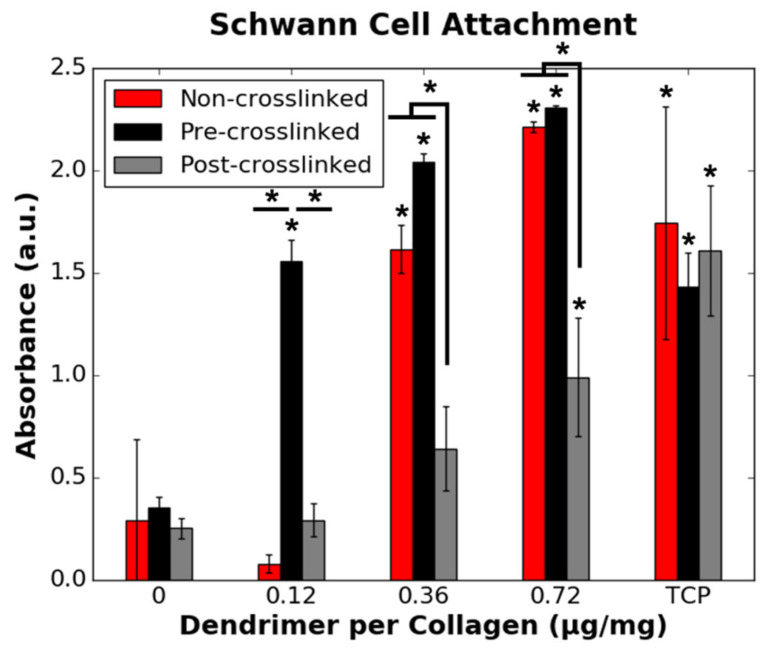
Effect of the quantity of IKVAV-capped dendrimers added to the various collagen films on RSC binding. RSCs were seeded onto non-collagen coated wells (TCP controls) on the same 48-well plates as each collagen substrate variety. * denotes statistically significant difference (*p* ≤ 0.05) between the data point annotated and the respective 0 µg/mg value for each collagen substrate, unless indicated otherwise.

While fibroblasts had similar binding affinity to collagen films, irrespective of both the degree of crosslinking and extent of dendrimer activation, RSCs only had low binding affinity to non-crosslinked and crosslinked collagen films with 0.12 µg/mg or no dendrimer activation (Figure 6). Increasing the dendrimer quantity increased RSC attachment to non-crosslinked and pre-crosslinked substrates. RSC attachment was also increased to a statistically significant extent on post-crosslinked collagen films, albeit to a lesser extent than for non-crosslinked and pre-crosslinked films. RSC binding to 0.12 µg/mg dendrimer coatings, which was the lowest concentration used, showed a particularly pronounced dependence on the underlying collagen where only the pre-crosslinked surface showed peptide-dependent cell attachment. These results show that, unlike HDF binding trends, RSC binding to collagen substrates depends on the extent of crosslinking of the material and also the manner in which the dendrimers are integrated onto the substrates.

### 3.5. RSC and HDF Proliferation on Dendrimer-Activated Collagen Films

**Figure 7:**
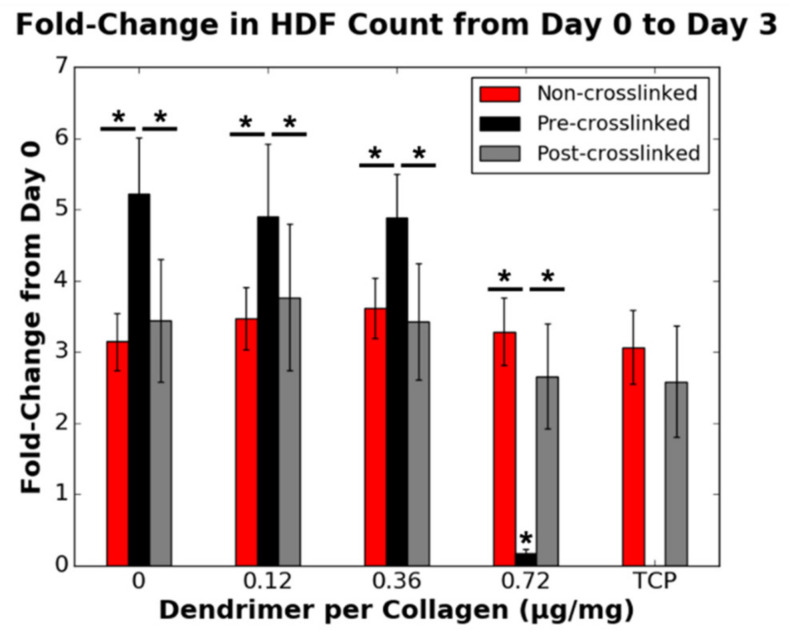
Effect of the quantity of IKVAV-capped dendrimers added to the various collagen substrates on the fold-change in HDF cell count from day 0 to day 3 (*n* = 9). A fold-change of 1 is equivalent to no change in cell density. HDFs were seeded onto non-collagen coated wells (TCP controls) on the same 48-well plates as non-crosslinked and post-crosslinked films. * denotes statistically significant difference (*p* ≤ 0.01) between the data point annotated and the respective 0 µg/mg value for each collagen substrate, unless indicated otherwise.

In addition to the cellular binding trends analysed after one day of incubation on the collagen substrates, cellular proliferation was probed after three-day incubation on the collagen surfaces. Cellular proliferation was calculated as a fold-change at day 3 from the initial loading density on day 0. A fold-change of 1 indicated no change in cell density. For non-crosslinked and post-crosslinked films, lower dendrimer quantities (0.12 and 0.36 µg/mg) did not reduce HDF proliferation relative to films without dendrimer activation (Figure 7). Despite the initially high extent of binding to pre-crosslinked collagen films, with 0.72 µg/mg dendrimer activation, HDF proliferation on these films was significantly reduced to below initial loading density after three-day incubation. HDF proliferation on post-crosslinked films was not statistically altered (*p* ≥ 0.05) by dendrimer activation. This data supports that dendrimer-activated collagen surface properties affect HDF proliferation, and that a relatively large quantity of untethered dendrimers integrated into pre-crosslinked films provides the desired ablation of fibroblast proliferation.

**Figure 8:**
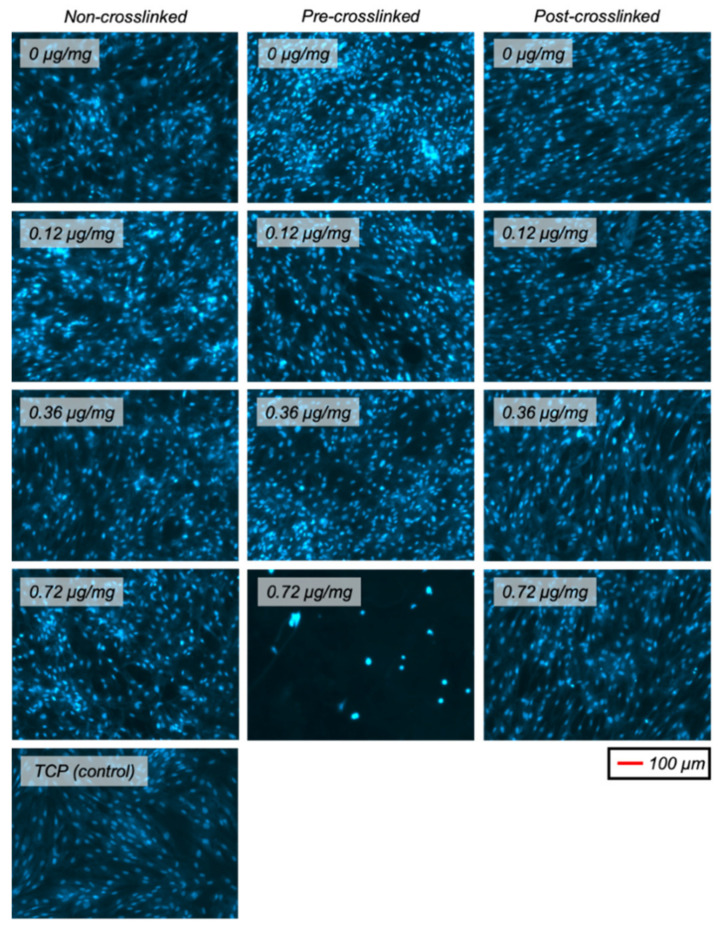
Representative fluorescent images (DAPI-stained nuclei) of HDFs on various collagen (or TCP) substrates after 3 days in culture.

DAPI-stained fluorescent images of HDFs after 3 days in culture were obtained to show the cell distribution (Figure 8). This showed that the cell distribution was similar for all samples, which all had uniform and non-clustered cell layers.

**Figure 9:**
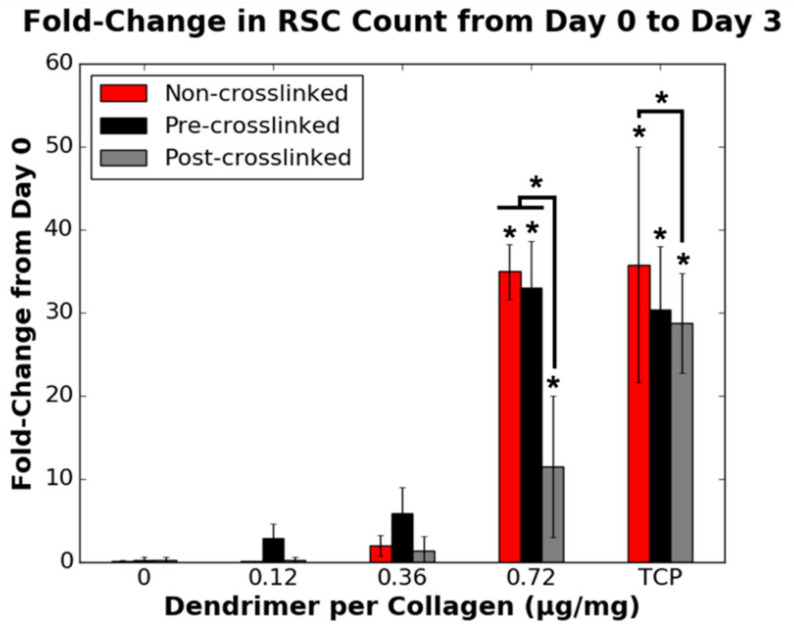
Effect of the quantity of IKVAV-capped dendrimers added to the various collagen substrates on the fold-change in RSC cell count from day 0 to day 3 (*n* = 9). A fold-change of 1 is equivalent to no change in cell density. RSCs were seeded onto non-collagen coated wells (TCP controls) on the same 48-well plates as each collagen substrate variety. * denotes statistically significant difference (*p* ≤ 0.001) between the data point annotated and the respective 0 µg/mg value for each collagen substrate, unless indicated otherwise.

RSC proliferation was dependent on the collagen substrate and peptide coating. Intermediate dendrimer coating concentrations (0.12, 0.36 µg/mg) increased RSC attachment to both non-crosslinked and pre-crosslinked films. However, only the highest dendrimer coating concentration (0.72 µg/mg) increased RSC proliferation, relative to non-dendrimer-activated films, for all three collagen substrate varieties (Figure 9). This increase was drastic for all three substrate varieties, reaching TCP positive control values, although the increase was observed to a lesser (~35%) extent for post-crosslinked films. These results support that dendrimer-activated collagen surface properties affect RSC proliferation. This data also points to an interesting parallel between HDF and RSC proliferation, wherein both cell types require a certain threshold quantity of IKVAV ligands to be present to modulate cell proliferation.

**Figure 10:**
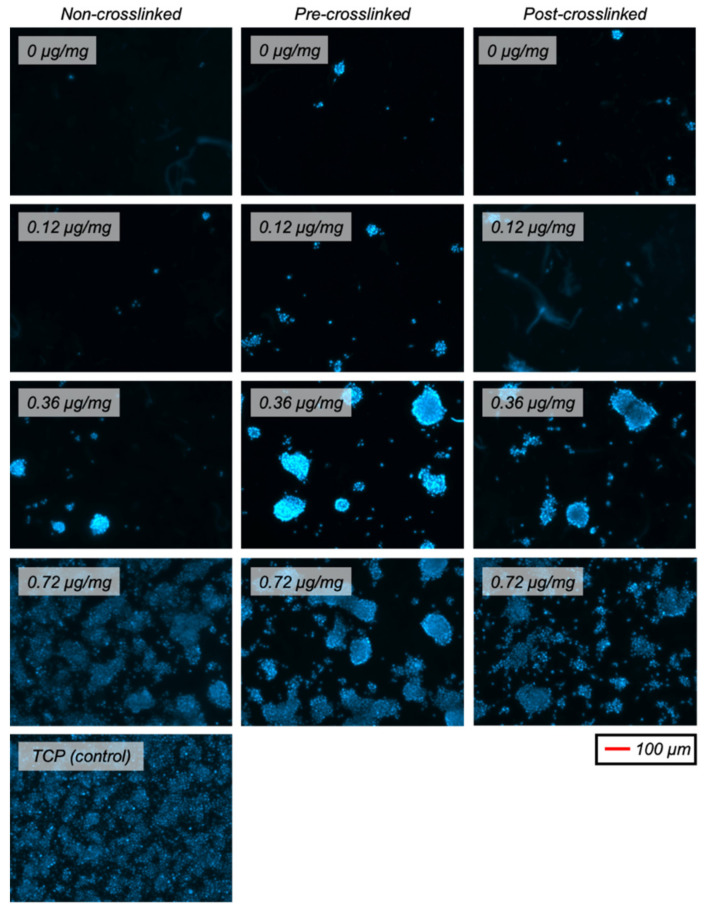
Representative fluorescent images (DAPI-stained nuclei) of RSCs on various collagen (or TCP) substrates after 3 days in culture.

As for HDF proliferation, DAPI-stained fluorescent images of RSCs on the different collagen substrates with various extents of dendrimer activation were obtained (Figure 10). Unlike HDFs, RSCs did not proliferate uniformly across the films and were rather clustered to certain areas of the substrates. This was especially evident for 0.36 µg/mg dendrimer coatings. Having low quantities of IKVAV-capped dendrimer, regardless of the extent of crosslinking, may provide insufficient affinity to RSCs on these collagen surfaces, and so cell-cell interactions dominate, leading to cell clustering. Conversely, with higher quantities of IKVAV motifs, the affinity of RSCs to these collagen surfaces may strengthen above cell-cell contacts, resulting in more uniform cell spreading. However, additional studies are required to understand why this cellular distribution occurs.

## 4. Discussion

### 4.1. Dendrimer-Collagen Associations and Long-Term Interactions

In this study, IKVAV-capped dendrimers were integrated into three substrate varieties: non-crosslinked collagen films, pre-crosslinked collagen films or initially non-crosslinked collagen films that were crosslinked after dendrimer addition (“post-crosslinked collagen films”). Prior to studying the cellular responses to these three substrate varieties, the chemical interactions between the dendrimers and the collagen films were probed.

It was predicted that dendrimers with higher MW and lower charge (78% dye-tagged dendrimers), due to their hydrophobicity and steric bulk, would preferentially associate with similarly hydrophobic, crosslinked collagen substrates. On the other hand, it was anticipated that dendrimers with lower MW and higher charge (8% dye-tagged dendrimers), which are more hydrophilic, and less sterically encumbered, would favour ionic or electrostatic associations with non-crosslinked collagen. However, both 78% and 8% dye-tagged IKVAV-capped dendrimers had higher initial association to post-crosslinked films than non-crosslinked films, even though both substrates were non-crosslinked at the point of dendrimer addition (Figure 2). Unlike post-crosslinked films, non-crosslinked films were initially incubated in 75% ethanol prior to dendrimer addition. Residual ethanol molecules may have interfered with dendrimer-collagen associations. Additionally, when an ion-rich solvent (PBS) was used to remove excess 8% dye-tagged dendrimers from non-crosslinked collagen films (Figure 2) prior to taking fluorescence measurements, few dendrimer-collagen associations remained. When excess dendrimers were instead removed with deionised water (Supplementary Materials, Figure S4), a high quantity of dendrimers remained associated to non-crosslinked films, on par with pre-crosslinked films for all but the highest dendrimer quantity. PBS, unlike deionised water, could disturb ionic dendrimer-collagen interactions, resulting in these observations. Together, these results suggest that initial dendrimer associations with non-crosslinked substrates are more variable than associations with crosslinked substrates as they can be hindered by local solute interference.

While the association studies suggested that dendrimer interactions with non-crosslinked films are dependent on local environments, the dissociation assays elucidated the dendrimer binding mechanisms for pre-crosslinked and post-crosslinked films, in addition to further supporting that dendrimers make ionic associations with non-crosslinked films.

For dendrimer-coated non-crosslinked films, the salt containing solutions, saline, PBS and 0.1% Tween (in PBS) dissociated a large quantity of 78% dye-conjugated dendrimers (Figure 3). It is likely that 0.1% Tween dissociated more dendrimers than other salt-based buffers as this buffer could additionally disrupt non-covalent dendrimer-collagen interactions with its detergent component. Surprisingly, ethanol, which is a non-ionic buffer, also dissociated dendrimers (*p* ≤ 0.001) to a significant extent. Ethanol may wash away non-covalently associated dendrimers like 0.1% Tween solutions. In addition, while water molecules stabilise collagen triple helices via hydrogen bonded hydration shells [30], larger ethanol molecules may form different hydrogen bonding interactions with non-crosslinked collagen. Different collagen surface environments promoted by ethanol washes may enable increased dendrimer dissociation. None of the various wash buffers significantly dissociated 8% dye-conjugated dendrimers from the non-crosslinked films (Figure 4), and so it is possible that the higher quantity of charged motifs on these dendrimers prevent full dissociation from the charged surfaces of non-crosslinked collagen. It is also possible that given the relatively low initial association to non-crosslinked collagen films, statistically significant differences in dissociation may be more challenging to detect than for 78% dye-tagged dendrimers. Overall, this data supports the theory that IKVAV-capped dendrimers interact ionically with non-crosslinked collagen films and also form some non-specific interactions.

Interestingly, citrate buffer (pH 3) did not significantly dissociate dendrimers from the non-crosslinked films. As an acidic buffer, citrate buffer was expected to increase dissociation of dendrimers from their collagen substrates as negatively charged glutamate (pKa 4.25) and aspartate (pKa 3.65) residues [31] become protonated at pH 3. Once protonated, these residues can no longer interact ionically with lysine (pKa 10.53) and terminal valine (pKa 9.62) residues [31] within IKVAV motifs, thus promoting dissociation. However, citrate buffer may be unable to dissociate dendrimers due to shielding of the dendrimer-collagen interface with an electrostatic double layer. This double layer may be formed with negatively charged citrate dihydrates in the citrate buffer and dendrimer-activated non-crosslinked substrates, which, due to the presence of IKVAV motifs, have exposed positive charges. Previous studies have shown that at mildly acidic pH (5.5 and 6.5), citrate is better than phosphate at reducing protein charge and double layer repulsion due to its multivalency at this intermediate pH [32]. However, in this study, because there are already charge-charge interactions at the collagen-dendrimer interface, close to the substrate surface, the additional electrostatic double layer provided by citrate may screen underlying ionic interactions between IKVAV-capped dendrimers and collagen films from further ion exchange. Because citrate dihydrates are much larger than chloride ions in saline, and phosphate ions in PBS or 0.1% Tween solutions, it is unlikely that they can penetrate through a layer of dendrimers to directly disturb dendrimer-collagen ionic interactions, rather forming an ionic screen. The effects of double layer electrostatic stabilisation may dominate over the effects of low pH for citrate buffer, resulting in dendrimer-collagen interactions remaining intact in citrate buffer solutions.

Furthermore, the dissociation assays also suggested that dendrimers are physisorbed onto pre-crosslinked films. Dendrimers, which are sterically bulky, likely entangle into pre-crosslinked substrates via weak non-covalent interactions, as these bioactive motifs quickly and completely dissociate when washed with any of the solvents. The lack of strong chemical interactions also explains why the dissociation of dendrimers from pre-crosslinked films is not dependent on the initial quantity of associated dendrimers (Figure 4). While 0.1% Tween and citrate buffer had statistically different dissociation patterns from the other wash buffers (*p* ≤ 0.05, not denoted in Figure 3 and Figure 4 for clarity), these deviations were minimal compared with the overall trend of immediate dissociation for all wash buffers. These results support the conclusion that while dendrimers are physisorbed onto pre-crosslinked films, these bioactive motifs quickly elute into aqueous environments.

**Figure 11:**
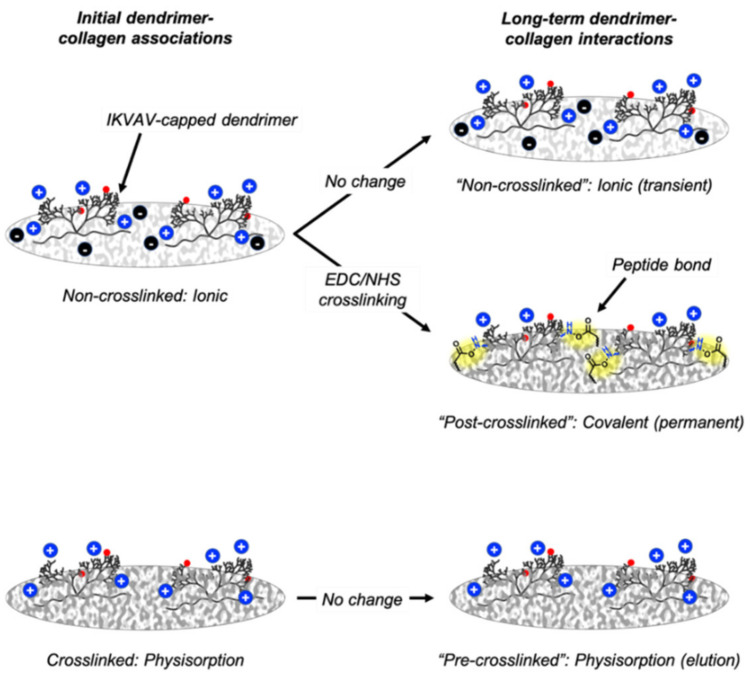
Chemical interactions between dendrimers and non-crosslinked, pre-crosslinked and post-crosslinked films. Red spheres depict Atto dye, blue spheres depict positively charged free amines on IKVAV-capped dendrimers, black spheres depict negatively charged glutamate/aspartate residues on non-crosslinked collagen, and yellow highlights dendrimer-collagen covalent bonds.

Finally, dendrimers may be more permanently bound to post-crosslinked films than either non-crosslinked or pre-crosslinked films as there was little dissociation for all wash buffers. The detergents used in these dissociation studies targeted ionic, non-ionic, and non-covalent interactions, showing that these are not the only bond types present between the peptide and collagen after EDC/NHS crosslinking. Although covalent interactions cannot be probed directly, as detergents such as SDS that disrupt all non-covalent interactions in proteins can also breakdown collagen films, these results suggest that covalent linkages may form between the peptide and collagen post-crosslinking. Like for non-crosslinked films, these stronger chemical interactions also explain why dissociation was proportionally increased when higher dendrimer quantities were initially integrated into the collagen films. These studies show that while EDC/NHS crosslinked collagen has been shown to have superior mechanical stability relative to non-crosslinked collagen, albeit at the expense of reduced bioactivity [21,22,33,34], when bioactivity is restored (i.e., by attaching IKVAV-capped dendrimers), these biochemical motifs only remain unequivocally integrated within the collagen films when they are permanently attached. The different dendrimer-collagen interactions are illustrated in Figure 11.

### 4.2. Effect of Dendrimer-Collagen Interactions on Cell Response

In designing biomaterials for neural applications, it is important to not only consider the response of neurite forming cells, but to also consider the responses of helper glial cells, such as Schwann cells, and inhibitory cells, like scar-forming fibroblasts. In this study, human dermal fibroblast (HDF) and rat Schwann cell (RSC) adhesion and proliferation on various IKVAV-capped dendrimer-activated collagen substrates were studied to better understand how various cell types interact with laminin-derived peptides, such as IKVAV, which have been thought to be promising for neural applications [12].

For biomaterials targeted at neural repair, the response of fibroblasts is extraordinarily important given that scar-forming fibrotic tissue is one of the greatest hinderances to attaining full-functional recovery after injury [5]. The data presented in this study supports the idea that initial HDF attachment to collagen is driven by the surface properties of collagen itself (Figure 5), while HDF proliferation is more likely to be affected by the presence of IKVAV biochemical motifs (Figure 7). While linear IKVAV peptides have been shown to minimise fibroblast adhesion on certain substrates [13], linear peptide AASIKVAVSADR coated onto tissue culture plastic was found to increase fibroblast adhesion at or above the mass of IKVAV used per sample in this study [35]. As shown in Figure 5, IKVAV-capped dendrimers also increase HDF adhesion to non-crosslinked collagen films with 0.36 or 0.72 µg/mg dendrimer activation. This provides evidence that the fibroblast response to IKVAV motifs may differ based on the type of substrate used, whether biological macromolecules or polymeric.

Although there was an increase in HDF adhesion to non-crosslinked films with greater dendrimer-activation, this increase was minimal relative to the already high affinity of HDFs to both non-crosslinked and crosslinked collagen films without dendrimer activation. When non-crosslinked collagen films, with or without IKVAV-capped dendrimers, are used, HDFs may bind exposed biochemical motifs on native collagen such as GFOGER, or laminin-derived peptides such as IKVAV, via β1-type cell-surface integrins [36]. This is from the perspective that expression of the β1 integrin has been shown to be necessary for dermal fibroblast adhesion, proliferation and migration [37] and GFOGER motifs on collagen specifically bind α1β1, α2β1, α10β1, and α11β1 integrins [26,36,38,39] while laminins can bind α1β1, α2β1, α3β1, α6β1, and α7β1 integrins [26,40]. HDFs may also non-specifically bind fully crosslinked collagen surfaces, which do not have exposed biochemical motifs, given their higher stiffness relative to their non-crosslinked analogues [29]. Previous studies have shown that EDC/NHS crosslinked collagen films are stiffer and have a higher tensile modulus than non-crosslinked collagen films [20]. Substrates with decreased elasticity have been shown to promote fibrosis *in vivo* [6] and human dermal fibroblasts have been shown to spread on stiffer collagen substrates [41]. While the tensile modulus of a dendrimer-activated collagen film is unknown, the dendrimer coating is a very thin, nanoscale layer on a substantially thicker micron scale collagen film. As the collagen film represents the overwhelming bulk of the material, it is likely that the dendrimer-layer contribution to the bulk mechanical properties would be minimal. Future integrin-based studies are necessary to determine if strong initial HDF binding to these materials is due to integrin-specific or non-specific bulk interactions, as IKVAV motifs have also been shown to interact with non-integrin cell-surface proteins [42]. However, the data presented here suggests that HDF adhesion to collagen, whether crosslinked or non-crosslinked, predominates over the effects of IKVAV biochemical signalling.

Unlike HDF adhesion, HDF proliferation, as well as both RSC attachment and RSC proliferation (proliferation defined here as fold-change in cell number over 3 days in culture), were affected not only by the surface properties of collagen but also the presence and quantity of IKVAV motifs present. As discussed above, dendrimers can bind ionically/non-specifically, non-covalently, or covalently to non-crosslinked, pre-crosslinked and post-crosslinked films, respectively. Ionic or non-specific interactions, and non-covalent physisorption do not alter the functional groups on IKVAV motifs. However, covalent binding reduces primary amines to amides on lysine or terminal valine residues on IKVAV. Higher RSC adhesion (Figure 6) and proliferation (Figure 9) on both non-crosslinked and pre-crosslinked films—for which the IKVAV motifs are unaltered—for higher dendrimer quantities suggests that it is necessary to have a high quantity of fully exposed, unaltered biochemical motifs to promote a change in cellular response. This aligns with the results shown in Figure 7, which show that HDF proliferation was only significantly lowered to below the initial loading density (fold-change <1) for pre-crosslinked films with 0.72 µg/mg dendrimer activation. It is likely that due to the higher quantity of inactivated IKVAV motifs on post-crosslinked films, the reduction in HDF proliferation cannot be detected on these substrates as this effect could be outweighed by the stiff nature of the crosslinked surface, which promotes HDF proliferation [41]. To reduce the cell count on crosslinked collagen substrates, which are optimal for HDF spreading, an abundance of fully exposed IKVAV motifs may be necessary.

When lower dendrimer quantities were used, there was lower than expected RSC attachment (*p* ≤ 0.05) and HDF proliferation on non-crosslinked films, relative to pre-crosslinked films, even though both substrate varieties had high quantities of unaltered IKVAV motifs. These lower-than-expected cellular responses may be affected by the decreased stiffness of non-crosslinked surfaces [29], which may be suboptimal for cell tractional forces and downstream cell-signalling. Poor initial attachment to these softer substrates may result in cell counts that are lower than the initial loading density after three days in culture, a deficit which can be compensated for by the presence of higher quantities of IKVAV motifs. For lower dendrimers quantities, the suboptimal effects of the non-crosslinked surface material may outweigh the desired effect invoked by the abundant presence of IKVAV motifs. Thus, when attaching nanoscale IKVAV-capped dendrimers to collagen surfaces, it is important that the properties of the material itself, such as extent of crosslinking, are optimised for cellular responses.

Overall, this study supports the hypothesis that fibroblasts and Schwann cells interact differently with mechanically stabilised and non-stabilised surfaces and shows that these cell types have different sensitivities to the quantity of IKVAV present on the collagen films. The initial binding profiles and subsequent proliferation trends also differ between the rat Schwann cells and human dermal fibroblasts investigated in this study. Thus, it is important to consider the optimal chemical environments for various cell types when designing materials for neural applications.

## 5. Conclusions

While neural guidance conduits are promising candidates for bridging small nerve gaps, more aptly designed biomaterials that reduce fibrosis are needed to address larger peripheral nerve injuries. This work has shown that when a high quantity of nanoscale IKVAV-capped dendrimers (0.72 µg/mg) was incorporated into pre-EDC/NHS crosslinked collagen films, rat Schwann cell attachment and proliferation were promoted while human dermal fibroblast proliferation was inhibited, as desired. These results show the potential of IKVAV-capped dendrimer-activated collagen biomaterials for neural applications since glial Schwann cells help neurite organisation and reduced fibroblast activity can limit detrimental fibrotic scarring at peripheral nerve injury sites. The different cellular responses to pre-crosslinked and post-crosslinked films also highlighted the importance of having fully exposed, non-covalently hindered biochemical motifs in attracting the desired cell type.

## Data Availability

The data for this article is available open access at https://doi.org/10.17863/CAM.68767.

## Supplementary Materials

The following are available online at https://www.mdpi.com/article/10.3390/nano11051157/s1, Figure S1–S3: collagen autofluorescence data, Figure S4: additional dendrimer association data.
