# Supplementary material for: Collagen Film Activation with Nanoscale IKVAV-Capped Dendrimers for Selective Neural Cell Response
Source: Nanomaterials (Basel). 2021 Apr 28;11(5):1157. doi: 10.3390/nano11051157 (PMC8146934; doi:10.3390/nano11051157)
Supplement: Supplementary file 1 [file nanomaterials-11-01157-s001.zip › nanomaterials-1187002-supplementary.pdf]

Collagen Film Activation with Nanoscale IKVAV-Capped Dendrimers for Selective Neural Cell Response

Jessica J. Kim, Daniel V. Bax, Robert Murphy, Serena M. Best * and Ruth E. Cameron *

Department of Materials Science and Metallurgy, University of Cambridge, 27 Charles Babbage Road, Cambridge CB3 0FS, UK; jessicajkim1@gmail.com (J.J.K.); dvb24@cam.ac.uk (D.V.B.); rm645@cam.ac.uk (R.M.)
* Correspondence: smb51@cam.ac.uk (S.M.B.); rec11@cam.ac.uk (R.E.C.)

Supporting Figures 1-3: For each wash buffer, there was minimal to no significant decrease in collagen autofluorescence over time for all three substrate varieties. This supported that non-crosslinked films were not significantly less stable than crosslinked films when incubated in aqueous wash buffers for more than seven weeks and supported that substrate breakdown was not a major confounding factor for the dissociation assays.

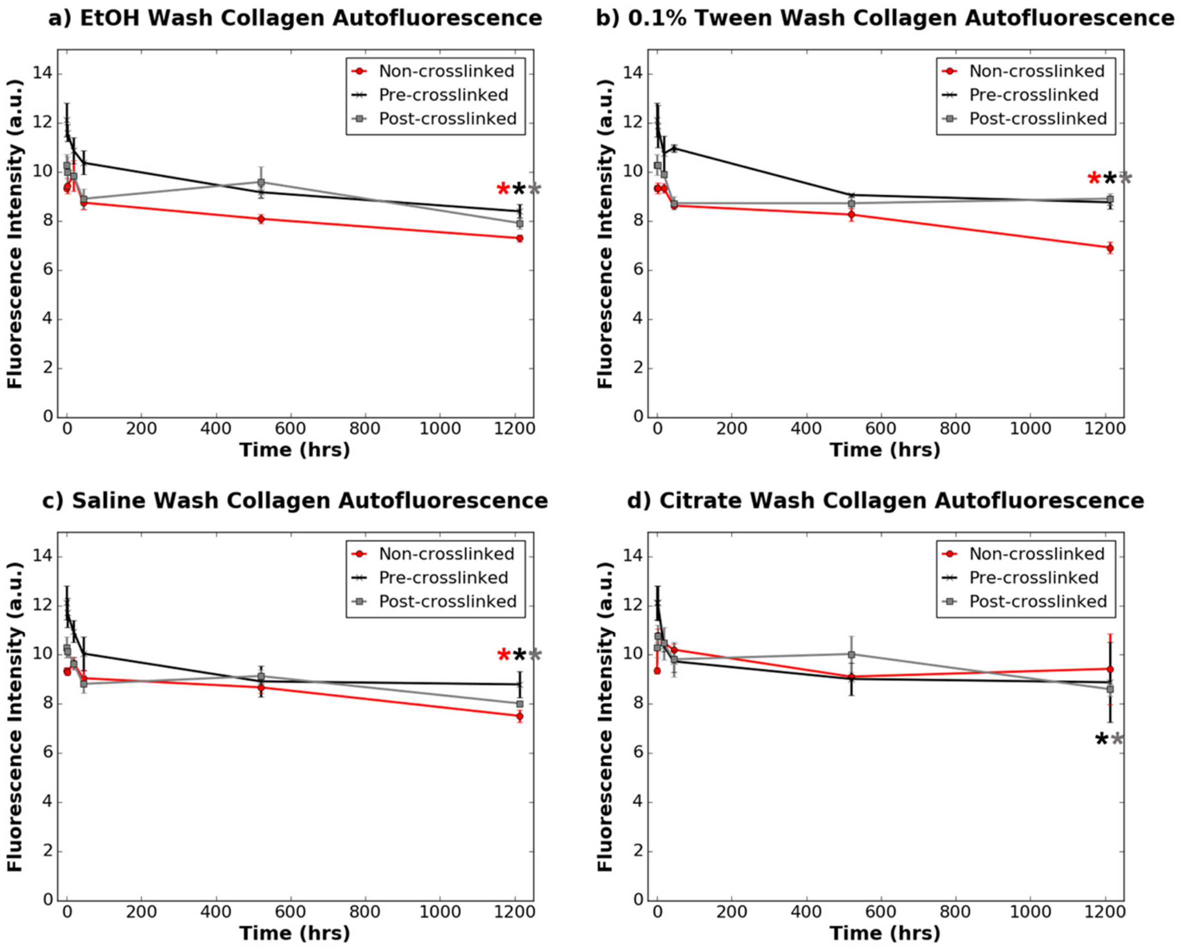

Figure S1. Collagen autofluorescence for 78% dye-tagged dendrimer (0.77 μg/mg loading concentration) dissociation from non-crosslinked, pre-crosslinked and post-crosslinked films. * denotes statistically significant difference ($p \leq 0.05$) between the data point annotated (colour-coded) and the respective 0 hr value for each collagen substrate.

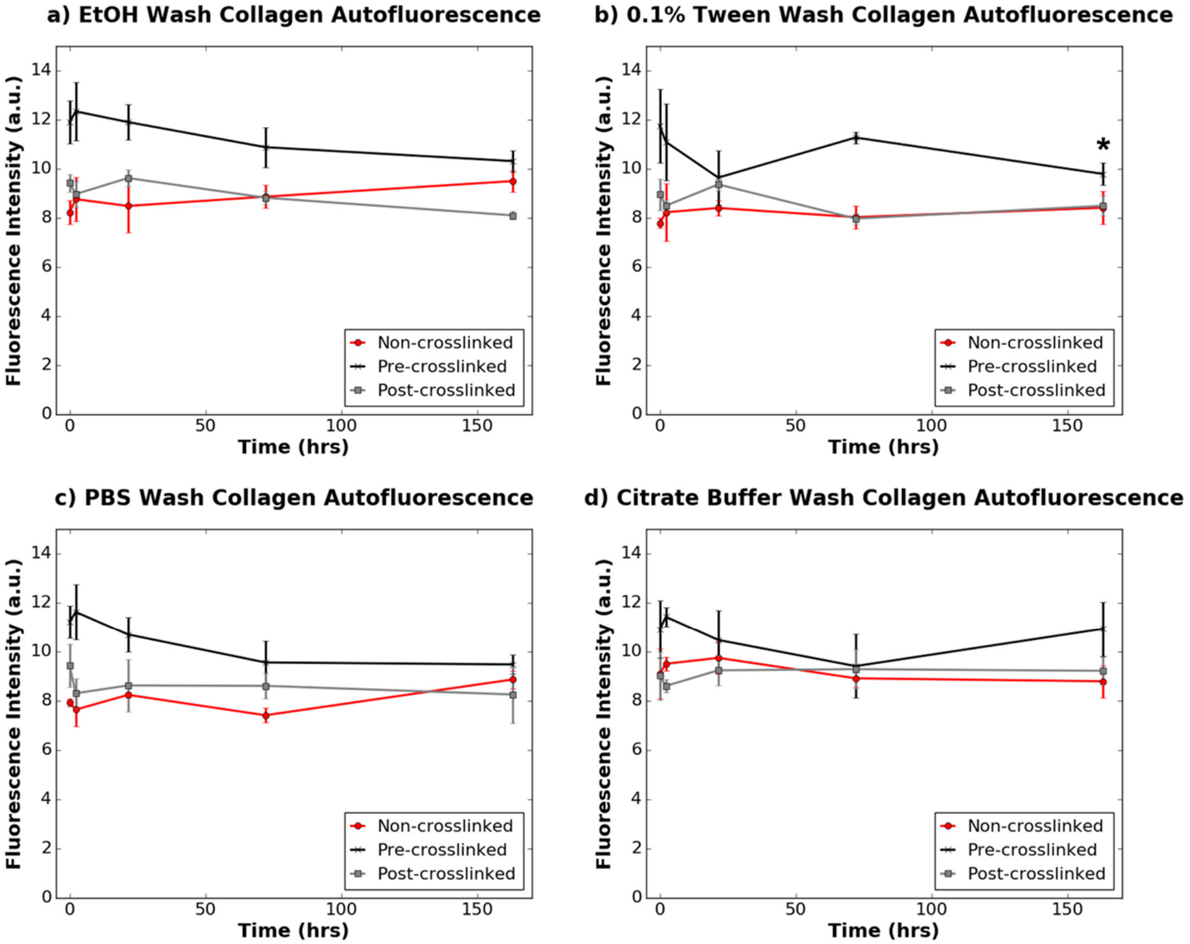

Figure S2. Collagen autofluorescence for 8% dye-tagged dendrimer (0.74 μg/mg loading concentration) dissociation from non-crosslinked, pre-crosslinked and post-crosslinked films. * denotes statistically significant difference ($p \leq 0.05$) between the data point annotated (colour-coded) and the respective 0 hr value for each collagen substrate.

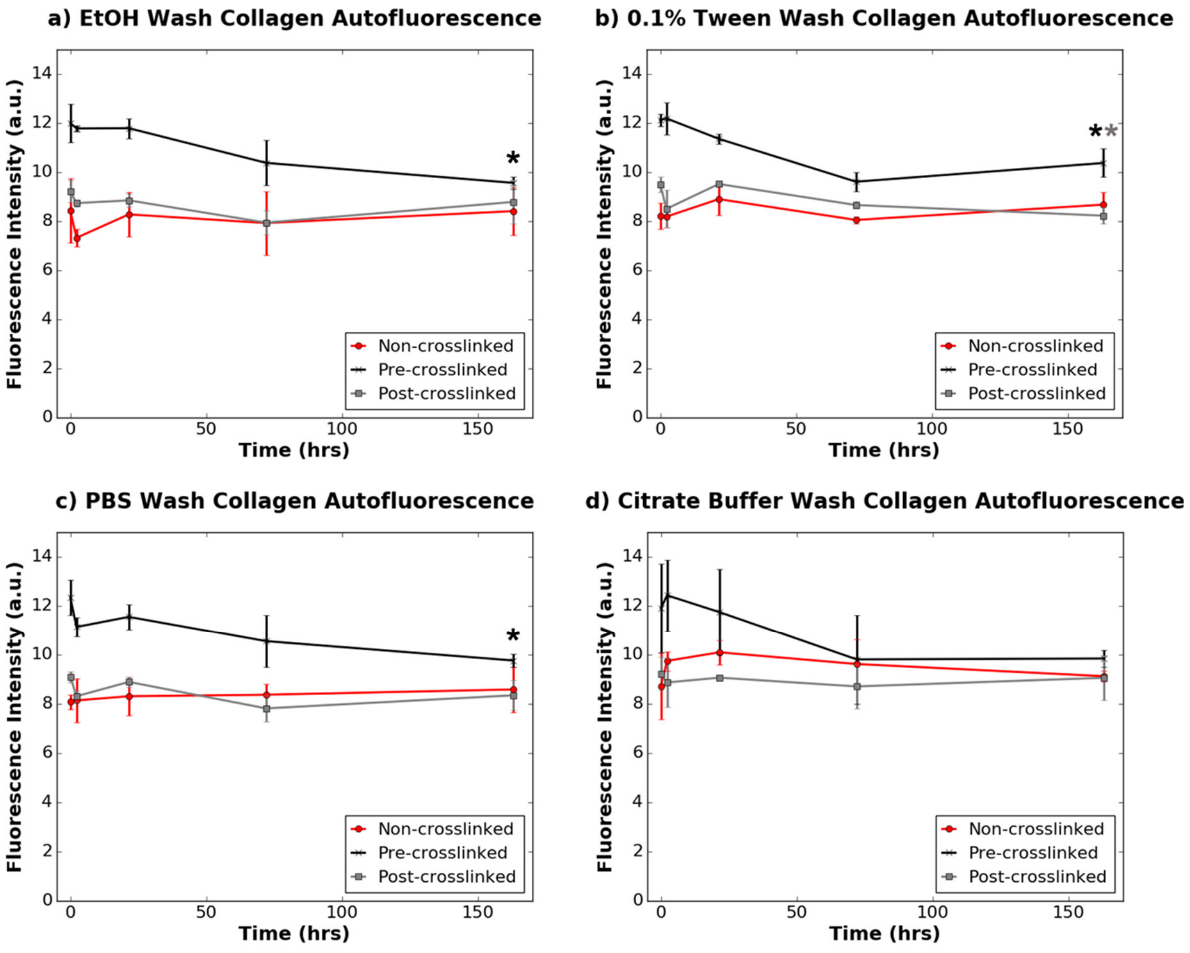

Figure S3. Collagen autofluorescence for 8% dye-tagged dendrimer (1.49 µg/mg loading concentration) dissociation from non-crosslinked, pre-crosslinked and post-crosslinked films. * denotes statistically significant difference ($p \leq 0.05$) between the data point annotated (colour-coded) and the respective 0 hr value for each collagen substrate.

Supporting Figure 4: When excess dendrimers were washed away with deionised water instead of PBS, the initial association to non-crosslinked collagen films was higher than that of pre-crosslinked films and on par with that of post-crosslinked films.

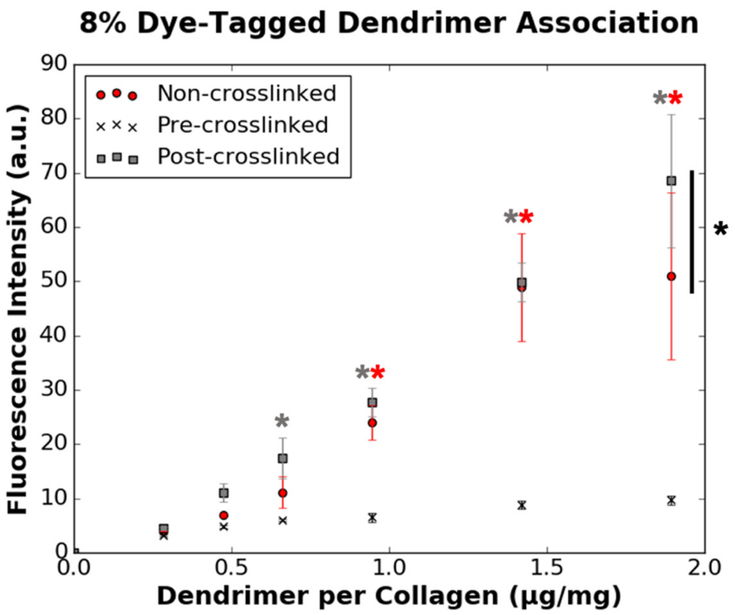

Figure S4. Effect of dendrimer quantity on the initial association of 8% dye-tagged dendrimers with the collagen films. * denotes statistically significant difference ($p \leq 0.05$) between the data point annotated (colour-coded) and the values for pre-crosslinked films with the same dendrimer quantity, unless indicated otherwise.
